# Anxiety, depression, and substance experimentation in childhood

**DOI:** 10.1371/journal.pone.0265239

**Published:** 2022-05-24

**Authors:** Robert J. Klein, Joseph A. Gyorda, Nicholas C. Jacobson

**Affiliations:** Geisel School of Medicine at Dartmouth College, Hanover, NH, United States of America; University of New South Wales, AUSTRALIA

## Abstract

Previous research has demonstrated that adults with comorbid depressive and anxiety disorders are significantly more likely to show pathological use of drugs or alcohol. Few studies, however, have examined associations of this type in children. A better understanding of the relationships between affective disorders and substance experimentation in childhood could help clarify the complex ways in which pathological substance use symptoms develop early in life. The present study included 11,785 children (M_age_ = 9.9) participating in the Adolescent Brain Cognitive Development (ABCD) study. Depressive and anxiety disorder diagnoses were evaluated as concurrent predictors of experimentation with alcohol and tobacco. A series of linear regressions revealed that children with either depressive or anxiety disorders were significantly more likely to experiment with alcohol or tobacco. However, children with both depressive and anxiety diagnoses were not more likely to experiment than children without a diagnosis. These results suggest that anxiety or depressive diagnoses in childhood may be associated with a greater likelihood of substance experimentation, but severe psychological distress may suppress these effects.

## Introduction

Substance abuse represents an enormous social, public health, and economic challenge across the globe [1, 2]. In the US alone, for example, a staggering 9.8% of all deaths are alcohol related [3], opioid use increased over 400% between 1999 and 2010 [4], and among youth, marijuana use disorder increased by an estimated 30% from 2008 to 2016 [5]. Meanwhile, according to the World Health Organization, the number of worldwide deaths attributable to drug abuse disorders increased by an alarming 47% between 2000 and 2016 [2]. From this perspective, substance use and abuse constitute a pressing public health challenge that should be met with enhanced scientific attention.

In addressing the societal burden of substance use, it may be desirable to develop strategies that address substance abuse across its developmental continuum, including prevention-oriented strategies targeting youth [6, 7]. In this domain, some theory-based interventions have shown promising results through targeting young people who may be at greater risk for future substance abuse [7–9]. Early-life risk factors have included early-onset substance use [9], living in high-risk family environments, or rebellious behavior in youth [7, 10]. Prevention-oriented interventions are desirable both because they represent an additional tool in the fight against substance use, and because they can be administered by parents, teachers, or trained volunteers [11], lessening the burden on overworked and outnumbered mental health professionals [12]. From this perspective, the more we know about key substance use risk factors and risk processes that manifest in young people, the better able we will be to design effective interventions and identify youth who need help the most [6].

One important substance use risk factor has been early onset of substance experimentation [13–16]. For instance, [14] showed that 11- to 12-year-olds who experimented with alcohol were (a) almost twice as likely to develop dependence later in life than youth who began experimenting at age 13–14, and (b) at over fifteen times greater risk than youth who began experimentation after age 18. Research investigating these processes in childhood has also shown associations between substance experimentation (in the form of sipping alcohol) and risk for future problematic alcohol consumption as early as age nine, even after controlling for parental risk profiles [16].

Critically, though, there is much to learn about features of childhood life or temperament that may impact childhood experimentation with drugs or alcohol. We propose that a more comprehensive knowledge concerning the specific childhood characteristics or processes that are associated with substance experimentation at this age would enhance our ability to both (1) design effective interventions to support youth who exhibit earl life substance use or experimentation, and (2) identify youth who are more likely to experiment with drugs or alcohol, thereby enabling timely support that could address risky substance use behavior before it has a chance to ruin lives. In sum, there is evidence linking early-life substance experimentation to substance abuse later in life, but more research is need to understand the processes and mechanisms that may impact this childhood experimentation.

In the present cross-sectional study, we examine a large representative sample of children to investigate the extent to which the presence of affective disorders early in life is associated with concurrent substance experimentation. We focus specifically on substance experimentation among children for two reasons. First, as we have said, experimentation with drugs or alcohol earlier in development is an important risk factor for future substance dependence. Second, we place an emphasis on substance experimentation because this behavior is present in childhood, while more significant substance use is not [17]. For instance, a large sample of 9–17-year-olds showed that only 1.2% of these youth had abused drugs or alcohol [18]. Thus, a potentially effective research approach for better understanding the developmental etiology of adult substance abuse may be to focus on the covariates—and potential drivers—of substance experimentation in childhood.

When considering early-life factors or processes that might contribute to experimentation with drugs or alcohol, it is useful to consider that substance abuse disorders in adulthood are typically preceded by affective pathologies such as depressive or anxiety disorders [19–24]. In addition, individuals who experience comorbid depressive and anxiety disorders tend to suffer greater emotional or affective distress as well as experience more severe substance use symptoms [25–27]. Thus, there is ample evidence that mental health problems related to negative affect may drive substance use in adults. These ideas are consistent with self-medication theories of substance abuse [24], as well as with transdiagnostic research and theory suggesting that dysregulated negative affect, such as intense sadness or anxiety, is a core diathesis across psychopathologies, including substance abuse [24, 28, 29]. Similar arguments have been proposed suggesting that difficulties with *managing* negative affect may drive substance abuse [29].

Importantly, there is some evidence of these links between excessive negative affect and substance use earlier in life. For instance, previous research has shown that substance use in teens has been mediated by a belief that substance consumption (smoking cigarettes) may reduce negative affect [30]. Relatedly, previous research has shown that a desire to escape negative feelings has mediated substance use as early as elementary school [31]. Thus, there are theoretical reasons to expect that significant depression or anxiety may represent important risk factors for substance consumption, even in children.

Although there is little direct evidence linking childhood pathological anxiety or depressive symptoms to concurrent substance use, childhood depressive or anxiety disorders have been linked with substance use problems later in life [32–34]. For instance, in a longitudinal study of 11-year-olds, internalizing disorders such as depressive disorders were predictive of substance use at age 14 [33]. Along these lines, childhood anxiety and depressive symptoms often co-occur [35], and, similar to adults with more serious mental illness, children who experience comorbid affective disorders could be at greater risk for developing early warning signs of substance use difficulties such as substance experimentation.

The aims of the present study are predominantly to examine the relationship between late childhood (e.g., age 9–12) affect-mediated disorders—both depressive and anxiety disorders—and concurrent substance experimentation. Here, given the prominence of self-medication theories of substance consumption [24], as well as links between substance use early in life and a desire to escape negative affect or emotions [30, 31], we hypothesized (1) that children with either a depressive or an anxiety disorder will be more likely to try alcohol or tobacco. We were also interested in the effect of co-occurring anxiety and depressive disorders on children’s tendency to experiment with substances. For reasons similar to the ones used to formulate our first hypothesis, we hypothesized (2) that children diagnosed with both depressive and anxiety disorders would be more likely to experiment with alcohol or tobacco than children with depressive or anxiety disorders alone.

Importantly, key features of the home environment may simultaneously influence both children’s mental health status and substance experimentation behavior. For this reason, the present study sought to control for confounds of this type. Among such environmental influences, parental socioeconomic status (SES) is perhaps the most salient. SES, for example, has been predictive of an array of environmental factors linked to substance experimentation such as parental support and monitoring [36] or the likelihood of being raised by a single parent [37]. Accordingly, the present models control for SES, as well as for gender and age of the child.

## Materials and method

### Participants and general procedures

The present study leveraged cross-sectional data drawn from the baseline assessment wave of the Adolescent Brain Cognitive Development (ABCD) study [38]. The ABCD study is an ongoing developmental project focusing on a nationally representative sample of 11,785 American youths and their parents. The present dataset was drawn exclusively from Wave 1 of the ABCD study, where participants were aged 9 to 12 years (M_age_ = 9.9, 47.9% female, 63.3% white, 15.7% African American, 7.2% Asian, 12.4% multiple races, and 1.3% other race; see Table 1). Of the participants, 1,720 were twins. Although we recognize that relying on cross-sectional data of this type presents important limitations, the goal of the present work was to examine the correlates of substance experimentation as early in life as possible. Thus, our optimal dependent variable was assessed at baseline only. This present ABCD baseline assessment dataset is publicly available to anyone upon request and can be accessed via the ABCD’s study’s website at: https://www.abcdstudy.org/scientists/data-sharing/.

**Table 1:** Descriptive statistics of children in the ABCD study (N = 11785).

	Descriptive Values
Age	M = 9.9, SD = .62
Gender	47.9% female
Race	63.3% white, 15.7% Black, 7.2% Asian, 13.7% other
Household Income	Median = 75-100k
Parental Education	M = 16.2 years of education
Anxiety Diagnosis	N = 385 (3.27%)
Depressive Diagnosis	N = 593 (5.03%)
Comorbid Diagnosis	N = 98 (0.83%)
Total Experimentation	M = .24, SD = .44
Alcohol experimentation	M = .23, SD = .42
Tobacco Experimentation	M = .01, SD = .09

The present cross-sectional data was collected between September 1, 2016 and August 31, 2018 at 21 sites across the United States. Recruitment procedures were designed to obtain a sample that closely approximates the national population demographics averages related to ethnicity, SES, sex, race, and neighborhood characteristics [38]. Participants were primarily recruited from elementary schools; however, approximately 10% of the sample was obtained using other strategies, such as referrals from the current sample, outreach to community activity groups, and publicly available mailing lists. For additional information about the ABCD project methods and study design, see abcdstudy.org or [38]. In addition, further details on the sample, as well as measure and compensation information, have been previously published [39, 40]. All participants agreed to take part in the study and informed consent was obtained from legal guardians. All procedures received ethical approval from the relevant local institutional review boards [38].

### Measures

#### Depressive and anxiety diagnoses

See Table 1 for descriptive statistics for all variables. Depressive Diagnosis (593 participants coded as diagnosed, or 5.03%) and Anxiety Diagnosis (385 participants coded as diagnosed, or 3.27%) predictors were dichotomous and assessed using an abridged computerized version of the Kiddie Schedule for Affective Disorders and Schizophrenia DSM-5 (KSADS-5). The KSADS-5 was administered and scored by computer and completed jointly by children and parents. This procedure has shown strong reliability relative to the standard KSADS interview [39]. Depressive disorders were coded as a 1 if children had a past diagnosis or current KSADS-5 diagnosis of major depressive disorder, persistent depressive disorder (dysthymia), or an unspecified depressive disorder, and as a 0 if none of these was present. For anxiety disorders, a value of 1 indicated having a past diagnosis or current KSADS-5 diagnosis of social anxiety disorder or generalized anxiety disorder (GAD), and a value of 0 indicated having no current or prior history of social anxiety disorder or GAD. No children in the present dataset had missing data for these diagnoses.

#### Substance experimentation

Experimentation was assessed using a computerized form of the Timeline Follow Back questionnaire [41]. Binary substance-specific scores were first calculated. For alcohol experimentation, youth received a 1 if they reported trying any form of alcohol (e.g., “…have you EVER TRIED … at any time in your life… [a sip of alcohol such as beer, wine, or liquor (rum, vodka, gin, whiskey”)]), and a 0 if they had not. Identically structured items assessed whether youth had “ever tried” a tobacco product, including a single “puff”. In total, 2656 children reported alcohol experimentation, 92 reported tobacco experimentation, and 56 reported trying both alcohol and tobacco. Finally, a single substance experimentation score was calculated for each youth that equaled the sum of both alcohol and tobacco experimentation (M = .24, SD = .44).

#### Substance consumption

Consumption of an entire alcoholic beverage or entire cigarette—as opposed to a sip or a puff—was also assessed with 2 items: “How many STANDARD DRINKS of ALCOHOL such as beer, wine, or liquor (rum, vodka, gin, whiskey) have you had in your life?” and “How many TOBACCO PRODUCTS have you used in your life? (e.g., # of [entire] cigarettes)”. Rates of total consumption were very low for alcohol or tobacco consumption (0.2% of children reported drinking a single beer or more at any time in their lives; 0.2% of children reported consuming an entire cigarette or more). Because these base rates were very low, these variables are included for descriptive purposes only and are not incorporated into formal analyses.

#### Control variables

Control Variables included sex, age, and parental socioeconomic status (SES). Sex was encoded using a “biological sex at birth” item. SES was calculated by standardizing and averaging each parent’s self-reported educational attainment (highest grade completed) and self-reported total household income.

## Results

Three initial linear regressions were calculated to model links between depressive disorders, anxiety disorders, and general substance experimentation in childhood. All outcome variables were a sum score of two binary substance experimentation variables (having tried alcohol or cigarettes). These three models were performed using the GLM function in R version 4.0.1 [42]. All models included control variables of household SES, age, and gender.

### Generalized substance experimentation

**Fig 1:**
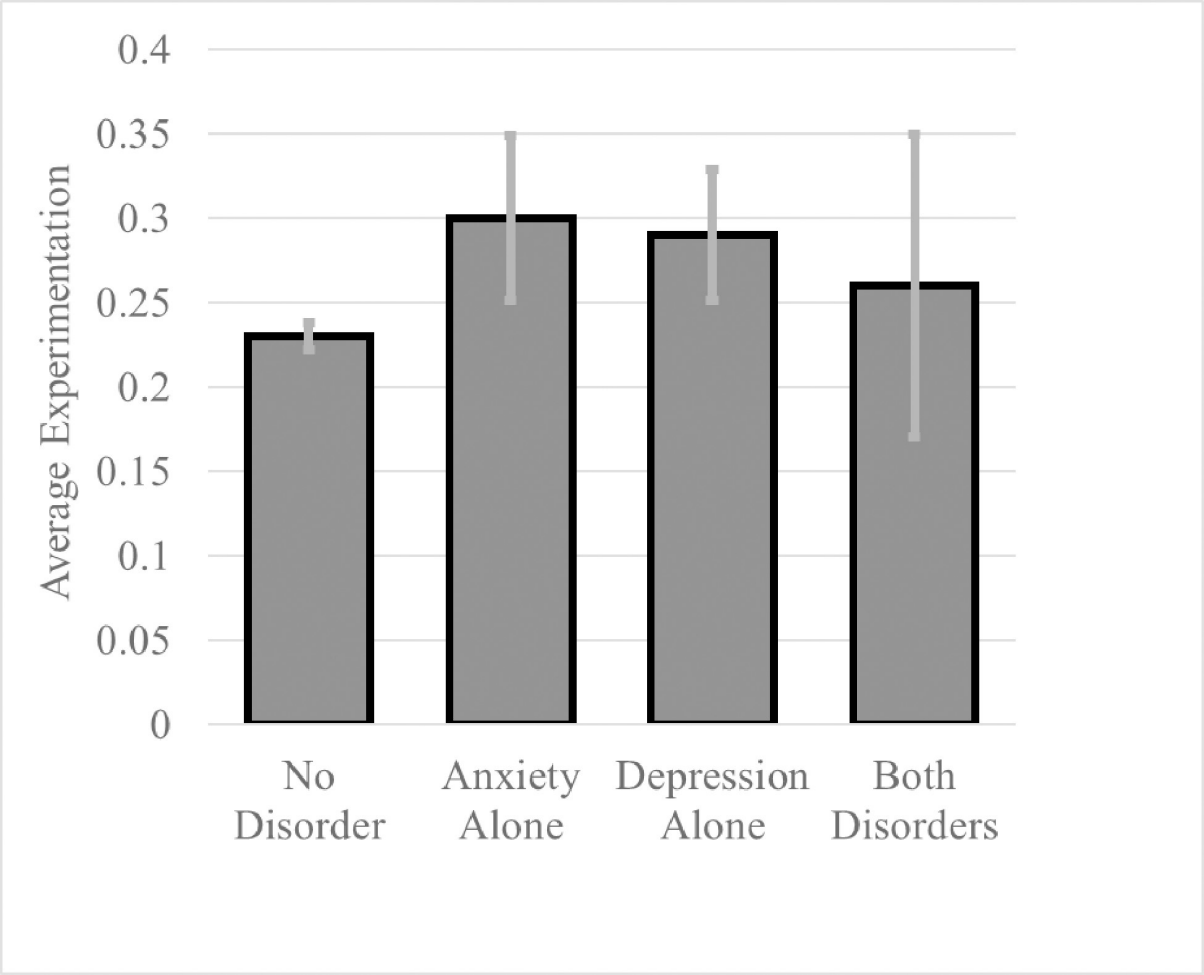
Mean substance experimentation as a function of diagnosis status. *Note*: Error bars represent 95% Confidence Intervals at alpha = .05.

The results of these three regressions can be seen in Table 2. In all models, both depressive and anxiety diagnoses were linked to greater substance experimentation among children. The final model also revealed a significant interaction between depressive and anxiety diagnoses. To understand these main and interaction effects, we calculated 95% confidence intervals for mean experimentation levels in all four possible diagnostic groups (no diagnosis, depressive, anxiety, and co-occurring depressive and anxiety diagnoses). These means and confidence intervals were plotted in Fig 1, which shows the extent to which having a depressive or anxiety diagnosis was associated with increased substance experimentation among children. In addition, Fig 1 reveals that children with co-occurring depressive and anxiety diagnoses were not more likely to experiment with substances than children without these diagnoses. This interaction finding contrasts with our hypotheses and suggests that children with comorbid anxiety and depressive disorders may be at lesser risk of trying substances compared to those with either an anxiety or depressive disorder.

**Table 2:** Three models examining affective diagnostic status as a predictor of generalized substance experimentation.

Predictor(s)	Outcome	*b*	*SE*	*t*	*p*
Model 1					
Depressive Diagnosis	Substance Experimentation	.08	.02	4.07	< .001
Model 2					
Anxiety Diagnosis	Substance Experimentation	.08	.02	3.29	< .001
Model 3					
Depressive Diagnosis	Substance Experimentation	.09	.02	4.18	< .001
Anxiety Diagnosis		.10	.03	3.53	< .001
Depressive x Anxiety		-.16	.06	-2.73	.006

*Note*: All models control for age, sex, and parent SES.

### Substance specific models

In two follow-up logistic regressions, we re-ran the previous interaction models but examined the binary alcohol experimentation and tobacco experimentation outcomes separately. The results of these models are summarized in Table 3. These models also included control variables of age, SES, and gender.

**Table 3:** Logistic regression follow-ups examining diagnostic status as a predictor of alcohol and tobacco experimentation separately.

Predictor(s)	Outcome	*Estimate*	*Odds ratio*	*SE*	*z*	*p*
Model 4						
Anxiety Diagnosis	Alcohol Experimentation	.46	1.58	.14	3.32	.001
Depressive Diagnosis	Alcohol Experimentation	.42	1.51	.11	3.83	< .001
Depressive x Anxiety	Alcohol Experimentation	-.76	.47	.31	-2.47	.014
Model 5						
Anxiety Diagnosis	Tobacco Experimentation	1.09	2.97	.47	2.31	.021
Depressive Diagnosis	Tobacco Experimentation	1.21	3.37	.33	3.66	< .001
Depressive x Anxiety	Tobacco Experimentation	-1.29	.28	.91	-1.42	.157

*Note*: All models control for age, sex, and parent SES.

The alcohol model results mirrored those of the previous generalized substance experimentation models. Logit coefficients indicated that both a depressive (odds ratio = 1.51, z = 3.83, p = .001) or an anxiety disorder (odds ratio = 1.58, z = 3.32, p = < .001) caused the model to expect significantly increased odds of trying alcohol, while the interaction coefficient was significant and negative (odds ratio = .47, z = -2.47, p = .014). Because this interaction coefficient equaled zero unless a participant had both disorders, this coefficient indicated that the presence of co-occurring disorders led the model to adjust significantly downward the odds of experimentation among children with co-occurring disorders. To directly quantify the magnitude of these effects, the “model predict” package in R was used to convert these coefficients to expected probabilities. This output showed that a) children without any diagnosis had approximately a 23% probability of experimentation, b) children with a depressive (28.2%) or anxiety (31.3%) disorder had an increased probability of experimenting, and c) that children with both disorders had expected probabilities more similar to participants without a diagnosis (24.7%). These results mirror the mean-level findings presented in Fig 1.

As shown in Table 3, the tobacco results also showed a significant increase in odds associated with depressive (odds ratio = 3.37, z = 3.66, p < .001) or anxiety disorders (odds ratio = 2.97, z = 2.31, p = .021). However, this model showed no significant interaction (odds ratio = .28, z = -1.42, p = .157). Directionally, this interaction effect mirrored the odds ratios in the previous models and also showed a larger relative magnitude. The coefficient did not reach significance, though, likely because of the much larger standard error in this model (see Table 3).

## Discussion

Within data drawn from the existing ABCD study, the present research examined ways in which affect-mediated disorders and substance experimentation interact early in development. The current findings suggest that children with either a depressive or anxiety disorder had an increased likelihood of experimenting with alcohol or tobacco. However, children with comorbid depressive and anxiety disorders were not more likely to experiment than children without either disorder (see Fig 1).

Previous research has shown concurrent links between mental health problems and substance use in adolescence [43], as well as links between early-life psychopathology and substance use disorders later in life [34]. The present results extend this work by demonstrating links between psychopathology and substance consumption occurring as early as age nine. To our knowledge, the present results show some of the earliest links between mental health and substance use. In addition, the present experimentation main effects may help clarify mechanisms by which early-life mental health problems may impact future substance abuse. For instance, substance experimentation early in life has been linked to greater risk of substance abuse later in life [15]. Thus, the current results should suggest to parents and caregivers who live with children experiencing mental health problems the importance of closely monitoring their children for early onset substance experimentation.

Also relevant to the present findings is the idea that, in adults, the causal or temporal relationship between affective psychopathology (e.g., depression) and substance-use disorders is rather murky and often characterized as bidirectional [44]. For instance, the psychological difficulties associated with affective disorders such as major depressive disorder can lead to escape-oriented substance use in adults [24], but life difficulties and stressors caused by substance abuse may also contribute to the development of psychopathologies [44]. However, given that the prevalence of *significant* substance consumption was very low in the current sample (e.g., just 0.2% of children reported drinking an entire beer in their lives), the present links between affective disorders and substance experimentation are not likely to originate from the life difficulties or stress caused by significant substance use. Thus, although the present findings cannot rule out third variables such as executive control or self-regulation ability impacting both substance experimentation and affective psychopathology, we can rule out the possibility that stress or life difficulties associated with addiction or heavy substance consumption are driving the present links between affective disorders and substance experimentation. From this perspective, the present main effects of depressive and anxiety disorder status may be in part due to some feature or byproduct of the affective disorder impacting the likelihood of substance exploration.

For adults, substance abuse may often be motivated by a desire to escape the psychological pain that is characteristic of affective disorders [24, 45, 46]. The present results suggest that this desire to self-medicate may also be present in children. For instance, older children may observe their parents or other role models address personal suffering with substances and thereby associate substance use with distress reduction. Such ideas are consistent with previous research showing that substance use in teens can be mediated by a belief that substance consumption (e.g., smoking cigarettes) may reduce negative affect [30]. Research has also shown that a desire to escape negative feelings can mediate substance use as early as elementary school [31]. Thus, there are theoretical and empirical reasons to expect that depressive and anxiety disorders may represent important risk factors for substance consumption, even in children.

Although our results show positive associations between affective disorders and substance experimentation and are thus consistent with existing data and theory, it is more difficult to explain the current suppressive interaction where children with either a depressive or anxiety disorder showed increased experimentation, while children with co-occurring disorders did not show a further increased experimentation. Adults with comorbid affective disorders, also known as serious mental illnesses (SMI), have shown higher rates of substance use disorders [25, 47]. However, we show the opposite pattern among children in the present study, where children with co-occurring disorders (or SMI) were not more likely to experiment with substances than mentally healthy children. Given our findings, as well as the previously discussed theoretical and empirical reasons to expect that experimentation in children is related to a desire to escape suffering, it becomes plausible that this experimentation suppression effect may be related to the different social contexts in which adults and children tend to use substances. Among adults, substance consumption outside of social contexts is a common and important risk factor for addiction, while youth appear to be unlikely to engage in substance use on their own [48]. However, children often experiment with drugs in social contexts [48], and often young people obtain substances via peer interaction [49]. In this way, substance experimentation in children may be a more socially modulated activity than substance use in adulthood.

Given the potential for social modulation of substance experimentation in children, one possible explanation for our suppression results could be that children experiencing SMI tend to interact with peers less frequently than children with depression or anxiety alone. In this way, children with SMI may be less likely to be socially exposed to drugs and alcohol. Myriad studies have linked early life mental illness severity to social isolation. In a study of fifth graders, for example, [50] found that children with comorbid depression and anxiety experienced impaired peer relations and social behavior. These children were also more withdrawn as well as socially rejected by peers [50]. Importantly, [51] reported that children with comorbid anxiety and depression were more socially withdrawn than children without SMI. Such findings are consistent with the notion that children with more severe mental health problems may have smaller social circles and experience more social rejection [52] and may therefore have fewer opportunities to experiment with substances than children with less severe mental illness. In sum, children with comorbid depression and anxiety may experience social isolation, leading to less socially mediated substance experimentation.

The present study has several strengths, such as a large representative sample, measurement techniques that have shown strong reliability, and an ability to examine links between affective disorders and substance use at quite early ages. A limitation of the present study, however, is our reliance on cross-sectional data. To study substance experimentation as early in childhood as possible, however, such a data structure was acceptable. It should be noted that although we have framed the present causal arrow between childhood psychopathologies and substance experimentation as originating—at least partially—from the emotional suffering associated with depressive and anxiety disorders, the current cross-sectional methods cannot rule out third variables, including risk factors that could simultaneously contribute to the development of both mental health problems and substance experimentation tendencies [53]. However, we show that our effects remain significant after controlling for SES, an environmental factor strongly associated with early life stress [54, 55].

## Conclusion

We present novel evidence for a positive association between mental health problems and substance experimentation in childhood, as well as some evidence of an attenuation of this association among children with the most severe psychological difficulties (i.e., comorbid depression and anxiety).
